# Editorial

**DOI:** 10.1365/s43439-021-00027-6

**Published:** 2021-04-07

**Authors:** Dennis-Kenji Kipker

**Affiliations:** Institut für Informations-, Gesundheits- und Medizinrecht (IGMR), Universität Bremen, Universitätsallee GW1, 28359 Bremen, Germany

## Dear reader,

More than a year has passed and the Corona pandemic still has us firmly in its grip. Familiar habits, vacations, conferences, trade fairs and meetings, and even the usual daily routine in the office—many things have been cancelled, and even more now take place purely digitally, currently with a still vague prospect of a return to normality. Compared to the state of digitization we had just a year ago, however, a lot has happened, and what was still the exception rather than the rule for many in the past has become the habit. However, the ubiquitous digitization of work and private life also shows us how vulnerable society is and that citizens must be able to rely on the supply of critical services and goods. If all resources function as normal, no one worries—but it is a different story if the Internet or telephone fail for half a day or, even worse, the electricity, gas or district heating supply stops working. At a time when people are increasingly retreating into the private sphere to live and work as a result of the pandemic, it must be ensured that this sphere offers all the resources needed for society and the economy to continue to function “remotely.”

However, this is not always the case, as the massive blackout in the USA in February of this year made clear: In the middle of winter, hundreds of thousands of households were cut off from the power supply and, in some cases, also from the water supply, since the transport systems and control electronics no longer functioned properly due to the extreme cold. Europe was also affected by power outages and significant voltage fluctuations at the beginning of 2021, with the real danger of a total blackout. Political demands were quickly made during this time to ensure security of supply for the population. However, this issue is not just about the power supply, but more generally about the protection of critical infrastructures—those facilities that are a mandatory prerequisite for the functioning of public life. This primarily includes the sectors of energy, food, finance and insurance, health, information technology and telecommunications, media and culture, government and administration, transport and traffic, as well as public water supply and wastewater disposal.

More and more—and not just since the start of the Corona pandemic—such essential facilities are connected to the digital data network and are therefore increasingly vulnerable from a cybersecurity perspective. Years ago, for example, simulated waterworks were operated that could be found on the Internet (so-called honeypots), which were deliberately compromised by attackers within just a few minutes. In this context, it was also proven, for example, that small and medium-sized water supply companies in particular have massive problems in adequately securing their critical systems against cyberattacks.

This is where the legislator comes into play, since guaranteeing not only the water and energy supply, but also the proper operation of all critical infrastructures is a central task of state regulation. For several years now, various approaches to this issue have been advocated in legislation around the world, many of which have similarities in terms of technical and organizational aspects, but which are often quite different in terms of their legal policy objectives. Since the end of last year, German and European legislators in particular have once again turned their attention to the legal regulation of IT security and especially of critical infrastructures. The European Union, for example, published its new cybersecurity strategy, “The EU’s Cybersecurity Strategy for the Digital Decade,” on December 16, 2020, and the draft for the new German IT Security Act 2.0 (IT-SiG 2.0) was the subject of considerable criticism by various experts in the committee meeting of the German Bundestag on March 1, 2021. All the more reason to take an in-depth look at the German draft law in this issue of the International Cybersecurity Law Review.

In addition to this article on the IT-SiG 2.0, the ICLR once again features numerous papers that shed light on cybersecurity regulation around the globe: An introduction to Korean cybersecurity law, the historical development of the cybersecurity framework in Japan, an introduction to Indian cybersecurity law, the regulation of digital space using China as an example, dealing with cybersecurity legislation in the United Kingdom for the post-Brexit period, the data security implications of the annulment of the EU–US Privacy Shield, and an assessment of the German implementation of the EU Digital Services Directive. Other interesting topics in this issue include dealing with COVID-19 and cybercrime from a practical perspective, the legal requirements for establishing an “open search engine,” how cybersecurity and outsourcing can be contractually coordinated, and the contractual requirements to be applied to pen testing. Also worth reading is the proposal for a new, comparative law concept of a “Cybersecurity Governance Triangle” in a European–US legal comparison.

With this issue of the ICLR, I would like to wish you many exciting and important insights as you start your legal journey around the world—and please do not hesitate to get in touch should you have feedback on any of the articles or an interesting topic of your own that you would like to write about!

With best wishes,

Dennis-Kenji Kipker

## Liebe Leserin, lieber Leser,

mittlerweile ist mehr als ein Jahr vergangen, und die Coronapandemie hat uns immer noch fest im Griff. Liebgewonnene Gewohnheiten, Urlaubsreisen, Konferenzen, Messen und Meetings und selbst der gewöhnliche Alltag im Büro – vieles wurde abgesagt, noch mehr findet mittlerweile rein digital statt – gegenwärtig mit einer noch unbestimmten Aussicht auf eine Rückkehr zur Normalität. Verglichen mit dem Stand der Digitalisierung, den wir noch vor einem Jahr hatten, ist jedoch viel geschehen, und das, was für viele in der Vergangenheit noch eher Ausnahme denn Regel war, wurde zur Gewohnheit. Die allgegenwärtige Digitalisierung von Beruf und Privatleben zeigt uns aber auch, wie verletzlich die Gesellschaft ist, und dass die Bürgerinnen und Bürger sich auf die Versorgung mit kritischen Diensten und Gütern verlassen können müssen. Wenn dabei alle Ressourcen wie im Regelfall funktionieren, macht sich niemand Gedanken – ganz anders jedoch, wenn einmal das Internet oder das Telefon für einen halben Tag ausfallen, oder, schlimmer noch, die Strom‑, Gas- oder Fernwärmeversorgung nicht mehr funktionieren. Wo man sich zum Leben und Arbeiten pandemiebedingt mehr und mehr in den privaten Raum zurückzieht, muss sichergestellt sein, dass dieser alle Ressourcen bietet, damit Gesellschaft und Wirtschaft weiterhin „remote“ funktionieren können.

Dass dies aber nicht immer der Fall ist, verdeutlichte jüngst, im Februar dieses Jahres, der massive Blackout in den USA: Inmitten des Hochwinters wurden Hunderttausende Haushalte von der Stromversorgung und teils auch von der Wasserversorgung abgeschnitten, weil aufgrund der massiven Kälte Transportsysteme und Steuerungselektronik nicht mehr richtig funktionierten. Auch Europa war zu Jahresbeginn 2021 von Stromausfällen und erheblichen Spannungsschwankungen betroffen, mit der realen Gefahr eines totalen Blackouts. Schnell wurden in dieser Zeit politische Forderungen laut, die Versorgungssicherheit der Bevölkerung zu gewährleisten. Bei diesem Thema geht es aber nicht nur um die Stromversorgung, sondern generell um den Schutz kritischer Infrastrukturen – also solcher Einrichtungen, die für das Funktionieren des öffentlichen Lebens zwingende Voraussetzung sind. Hierzu gehören vornehmlich die Sektoren Energie, Ernährung, Finanz- und Versicherungswesen, Gesundheit, Informationstechnik und Telekommunikation, Medien und Kultur, Staat und Verwaltung, Transport und Verkehr sowie die öffentliche Wasserver- und Abwasserentsorgung.

Mehr und mehr – und nicht erst seit Beginn der Coronapandemie – sind solche wesentlichen Einrichtungen an das digitale Datennetz angebunden und damit auch unter Gesichtspunkten der Cybersicherheit zunehmend vulnerabel. Schon vor Jahren wurden beispielsweise simulierte Wasserwerke betrieben, die im Internet auffindbar waren (sog. Honeypots), die schon nach wenigen Minuten vorsätzlich von Angreifern kompromittiert wurden. Nachgewiesen wurde in diesem Zusammenhang z. B. auch, dass gerade kleine und mittelständische Betriebe der Wasserversorgung massive Probleme haben, ihre kritischen Systeme hinreichend gegen Cyberangriffe abzusichern.

An dieser Stelle tritt der Gesetzgeber auf den Plan, denn nicht nur die Wasser- und Energieversorgung, sondern die Gewährleistung des ordnungsgemäßen Betriebs sämtlicher kritischer Infrastrukturen ist eine zentrale Gewährleistungsaufgabe staatlicher Regulierung. Bereits seit mehreren Jahren werden hierzu in der Gesetzgebung weltweit verschiedene Ansätze vertreten, die in technisch-organisatorischer Hinsicht vielfach Ähnlichkeiten aufweisen, aber in ihrer rechtspolitischen Zielsetzung nicht selten durchaus verschieden sind. Vor allem der deutsche und der europäische Gesetzgeber wandten sich seit dem Ende des vergangenen Jahres wieder verstärkt der gesetzlichen Regulierung von IT-Sicherheit (IT: Informationstechnologie) und speziell von kritischen Infrastrukturen zu. Die EU (Europäische Union) beispielsweise veröffentlichte ihre neue Cybersicherheitsstrategie *„The EU’s cybersecurity strategy for the digital decade“* am 16.12.2020, und der Entwurf für das neue deutsche IT-Sicherheitsgesetz 2.0 (IT-SiG 2.0) wurde in der Ausschusssitzung des Deutschen Bundestages am 01.03.2021 von verschiedenen Sachverständigen erheblich kritisiert. Umso mehr ein Grund dafür, in dieser Ausgabe des „*International Cybersecurity Law Review*“ einen vertieften Blick in den deutschen Gesetzentwurf zu wagen.

Neben diesem Beitrag zum IT-SiG 2.0 finden sich im *ICLR* auch dieses Mal wieder zahlreiche Beiträge, die die Regulierung der Cybersicherheit rund um den Globus beleuchten: eine Einführung in das koreanische Recht der Cybersicherheit, die historische Entwicklung des „cybersecurity framework“ in Japan, eine Einführung in das indische Recht der Cybersicherheit, die Regulierung des digitalen Raums am Beispiel von China, der Umgang mit Cybersicherheitsgesetzgebung im „United Kingdom“ für die Zeit nach dem Brexit, die datensicherheitsrechtlichen Folgen der Nichtigerklärung des „EU-US Privacy Shield“ und eine Bewertung der deutschen Umsetzung der Richtlinie für digitale Dienste der EU. Interessante Themen dieser Ausgabe sind darüber hinaus der Umgang mit COVID-19 („coronavirus disease 2019“) und Cyberkriminalität aus praktischer Sicht, welche rechtlichen Anforderungen für die Etablierung einer „open search engine“ gelten, wie Cybersicherheit und Outsourcing vertraglich abgestimmt werden können und welche vertraglichen Anforderungen an Pentesting anzulegen sind. Lesenswert ist außerdem der Vorschlag für ein neues, rechtsvergleichendes Konzept eines „cybersecurity governance triangle“ im europäisch-US-amerikanischen (US: „United States“) Rechtsvergleich.

Auch für diese Ihnen vorliegende Ausgabe des *ICLR* wünsche ich Ihnen viele spannende und wichtige Erkenntnisse bei der juristischen Reise rund um die Welt – und zögern Sie nicht, den Kontakt aufzunehmen, sollten Sie Feedback zu einem der Beiträge oder selbst ein interessantes Thema haben, über das Sie gerne schreiben möchten!

Mit den besten Wünschen und alles Gute,

Dennis-Kenji Kipker

